# Study of necrotic apoptosis by pulsed electric field ablation in rabbit left ventricular myocardium

**DOI:** 10.3389/fcvm.2022.1012020

**Published:** 2022-09-26

**Authors:** Zhihong Zhao, Yonggang Chen, Bin Wu, Gaodong Qiu, Liangjie Hong, Xinhua Chen, Xingwei Zhang

**Affiliations:** ^1^Department of Cardiology, The Affiliated Hospital of Hangzhou Normal University, Hangzhou, China; ^2^Key Laboratory of Pulsed Power Translational Medicine of Zhejiang Province, Hangzhou, China; ^3^Hepatobiliary and Pancreatic Surgery, The First Affiliated Hospital of Zhejiang University, Hangzhou, China

**Keywords:** cardiovascular pathology, rabbit, heart, ablation, pulsed electric field, necrotizing apoptosis

## Abstract

**Objective:**

We investigate the characteristics of histological damage to myocardial cells in the ablation region and surrounding areas of the left ventricular epicardium in rabbits using our self-developed cardiac pulsed electric field (PEF) ablation instrument and ablation catheter.

**Methods:**

Forty eight New Zealand rabbits underwent ablation on the left ventricular myocardium after open-heart exposure with a cardiac arrhythmia PEF ablation device and ablation catheter developed by the Medical Translation Laboratory of Pulsed Electric Field Technology in Zhejiang Province. The ablation parameters were set as biphasic electrical pulses; voltage, ±800 V; pulse width, 10 μs; interphase delay, 500 us. Six rabbits were included in the sham group and 42 other rabbits were randomly divided into immediately, 6-h, 1-, 3-day, 1-, 2-, and 4-week post-ablation groups, with six rabbits in each group. Creatine kinase- (CK)-MB isoenzyme (CK-MB), aspartate aminotransferase (AST), and lactate dehydrogenase (LDH) levels were measured before and at different time points after PEF ablation to analyze their dynamic evolution. Masson staining of tissue block sections of left ventricular myocardial ablation and adjacent tissue heart specimens was performed, and the occurrence of TUNEL apoptosis in myocardium tissue was analyzed.

**Results:**

All rabbits completed the PEF ablation procedure and the follow-up process. After PEF ablation, the levels of cardiac enzymes, including CK-MB, CK, and AST, increased significantly, peaking 1–3 days after the procedure. In particular, those of CK and CK-MB increased by 15–20 times but returned to the preoperative level after 2 weeks. Based on general observation, it was found that the myocardium in the ablation area was swollen immediately after PEF ablation. Masson staining analysis revealed that cardiomyocytes were broken and infiltrated by erythrocytes after 6 h. After 1 day, the cells started to experience atrophy and necrosis; after 3 days, fibrotic replacement of the necrotic area became obvious. Then, by 4 weeks, the myocardial cells were completely replaced by hyperplasia. Apoptosis occurred significantly at 6 h and peaked at 24 h post-ablation, demonstrating a 37.7-fold increase; apoptotic cell counts decreased significantly at 3 days post-ablation, and no significant apoptotic cardiomyocytes were seen after 1 week.

**Conclusion:**

After PEF ablation, cardiomyocytes showed apoptotic process and dyed, at least partially, through a secondary necrosis, the ablation boundary was clear, the ablation area was replaced by structurally intact fibroblasts, no island myocardium tissue were seen, and the ablation area vessels and nerves were not affected.

## Introduction

Pulsed electric field (PEF) ablation is performed *via* transient, high-intensity pulses that release extremely high electric field energy, resulting in irreversible electroporation followed by cell death. The evolution of cell death after electroporation is key to a comprehensive understanding of the effects of irreversible electroporation (1), and the selection of the optimal pulse ablation protocol (2). The irreversible process of tissue cell death by electroporation includes necrosis/cell death, apoptosis/cell death, necroptosis/programmed necrosis, and apoptosis/inflammatory necrosis (3). Recently, a domestic Chinese PEF ablation system for paroxysmal AF ablation was reported (4). The process and evolution of myocardial tissue death by irreversible electroporation have not yet been detailed (5). In this study, a novel PEF ablation system was developed based on the medical–industrial combination of the previous multidisciplinary frontier crossover innovation team, and, using this instrument, PEF ablation was performed in rabbit hearts. Subsequently, the effects on myocardial cell histology at different time points after ventricular ablation were continuously observed to analyze the clinical application of the PEF ablation system.

## Materials and methods

### PEF ablation device

**Figure 1:**
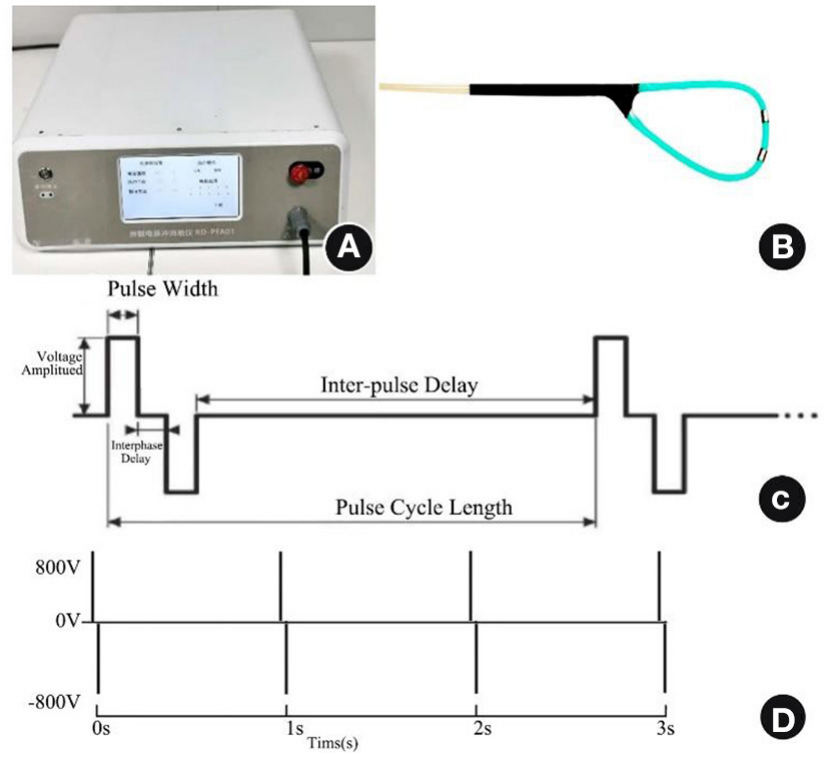
PEF ablation instrument, ablation electrode catheter, and PEF ablation parameters. **(A)** PEF ablation instrument. **(B)** PEF ablation electrode under direct epicardial vision. **(C)** Biphasic PEF ablation parameters. **(D)** Fifty to one hundred pulse numbers delivered at a frequency of 1 Hz. PEF, Pulsed electric field.

The arrhythmia PEF ablation device developed by Zhejiang Pulsed Electric Field Technology Medical Translation Laboratory emits positive and negative biphasic pulses. To avoid triggering ventricular fibrillation by high-voltage electrical stimulation, this device adopts the technique of cardiac synchronization control; high-voltage ultrashort pulses are discharged during the cardiac effective non-response period, i.e., the absolute ventricular non-response period. The number of pulses delivered determines the intensity of ablation. The ablation electrode is a homemade electrode with an electrode ring made of platinum–iridium alloy and fixed to an outer tube made of Pebax (Arkema, Colombes, France). Its diameter is 1.22 mm, its length is 2 mm, and the distance between the two electrodes is 4 mm. The electrodes are used for direct contact ablation with the heart under direct epicardial vision; the electrode connection cable is connected to the interface of the PEF ablation instrument. The ablation parameters were set as biphasic electrical pulses; voltage amplitude ±800 V; pulse width 10 μs; interphase delay 500 us, cycle length 1 Hz, 50–100 pulse numbers to cause a sufficient ablation area (see Figure 1).

### PEF ablation of the rabbit heart

Male and female normal-grade adult healthy New Zealand White rabbits were purchased from Hangzhou Yuhang KeLian Rabbit Professional Cooperative (SCXK Zhe2017-0004), weighing 2.5–3.5 kg. They were housed at the Zhejiang Experimental Animal Center (SYXK Zhe 2019-0011). Each surgery was performed with 3% sodium pentobarbital at 30 mg/kg after intravenous injection of anesthetics *via* the ear margins of the rabbits, and intravenous injection of 1,000 U of heparin for anticoagulation. After the rabbit was anesthetized and routinely fixed in the supine position on the animal operating table, an open-heart procedure was performed along the mid-thorax, the PEF ablation catheter was placed against the left ventricular myocardium of the heart for PEF ablation, no ablation procedure in the sham group. Six rabbits were included in the sham group and 42 other rabbits were randomly divided into immediately, 6-h, 1-, 3-day, 1-, 2-, and 4-week post-ablation groups, with six rabbits in each group. The procedure was performed strictly according to the product instructions, and the rabbits were reared after awakening. The study passed the ethical review of experimental animal welfare by Zhejiang Animal Center (approval no. ZJCLA-IACUC-20020024) and was conducted by a professional physician with an animal experimental qualification certificate.

### Myocardial enzyme

In addition to the six rabbits in the 4-week group, 10 rabbits were randomly selected, and 5 mL of blood was taken from each rabbit's ear marginal vein before PEF and 1, 2, 3 days, 1, 2, and 4 weeks after PEF and then placed in a separator/procoagulant tube. Creatine kinase- (CK)-MB isoenzyme (CK-MB), aspartate aminotransferase (AST), and lactate dehydrogenase (LDH) assays were performed. The principle of each assay was chemiluminescence combined with immuno-enhanced assay for batch detection, and the procedure was performed according to the kit (Lumigenex Co., Ltd, Suzhou, China) instructions.

### Histopathology of PEF-ablated myocardium and myocardial apoptosis

In the control sham-operated group, cardiac specimens were collected immediately, 6 h, 1, 3 days, 1, 2, and 4 weeks after ablation for Masson staining analysis, and apoptosis analysis of myocardium tissue in the ablation area was performed immediately, 6, 24 h, 3 days, and 1 week after ablation. Six rabbit specimens were taken from each group. The left ventricle was sliced longitudinally and transversely in successive 5-μm-thick sections, and Masson staining was performed on every fifth slide (the slides were treated with egg white glycerol beforehand). The results of Masson trichrome staining were as follows: blue (double staining) or green (double staining) for collagen fibers, red for myofibers and fibrils, and orange for erythrocytes, the images were taken in bright field mode of fluorescent microscope (NIKON, Eclipse Ci-E). We also used TUNEL Apoptosis Assay Kit (Yeasen Biotechnology Co., Ltd. Shanghai, China) following the manufacturer's instructions, the above pathological sections were simultaneously subjected to terminal deoxynucleotidyl transferase (TdT) dUTP nick-end labeling (TUNEL) apoptosis assay. Paraffin sections were dewaxed and repaired by phosphate-buffered saline (PBS) (pH, 7.4), covered by drops of film-breaking working solution, and incubated at room temperature; additionally incubated for 2 h with appropriate amounts of reagent 1 (TdT) and reagent 2 (dUTP) from the TUNEL kit, washed with PBS, and incubated at room temperature with drops of 4′,6-diamidino-2-phenylindole staining solution while avoiding light; washed with PBS, incubated with DAPI staining solution dropwise at room temperature avoiding light, and slightly shaken dry; sealed with anti-fluorescence quenching sealer; observed under a fluorescence microscope, and acquire images. DAPI emission from all cell nuclei and fluorescein emissions from positive apoptotic cell nuclei were detected on a fluorescent microscope using DAPI and FITC filter cubes, respectively. We counted the number of positive cells and total cells in five randomly selected fields of view. The apoptosis rate is calculated as follows: the number of positive cells/the total number of cells × 100%.

### Statistical analysis

Statistical analysis was performed using the SPSS version 20.0 software program (IBM Corporation, Armonk, NY, USA). Measurement data conforming to the normal distribution were expressed as $\bar{x}\;\pm$ SD, and the count data were described by frequency (composition ratio). A *t*-test was used to compare the measurement data between groups. Comparisons of count data between groups were performed using the Chi-squared test or Fisher's test. Differences were considered significant at *P* < 0.05.

## Results

Forty-eight rabbits completed the PEF ablation process and follow-up procedure in this study, including six rabbits in the control sham-operated group. During the PEF ablation procedure, the rabbits breathed smoothly, and the electrocardiogram recordings were smooth, without muscle twitching.

### Cardiac enzyme profile level

The cardiac enzymes were all significantly elevated at 1 day post-ablation. CK peaked 18 times higher at 1 day post-ablation, decreased significantly after 3 days, and was 2.5 times higher than the preoperative level at 4 weeks post-ablation. CK-MB peaked 13 times higher at 3 days post-ablation, decreased significantly after 3 days, and returned to the preoperative level at 14 days post-ablation. AST peaked twice higher at 1 day post-ablation, then decreased, increased again at 7 days post-ablation, and returned to the preoperative level at 4 weeks post-ablation. Finally, LDH peaked 6 times higher at 1 day post-ablation, then decreased, rose again to 10 times the preoperative level at 3 days post-ablation, then started to decrease again, and was 1.5 times higher than the preoperative level at 4 weeks post-ablation, as shown in Table 1.

**Table 1:** Myocardial enzyme profile levels at different time points before and after PEF ablation in rabbit hearts.

	***n***	**CK**	**CK-MB**	**AST**	**LDH**
Pre-Ablation	10	233.8 ± 44.7	220.4 ± 52.9	9.8 ± 4.8	40.7 ± 15.8
1 d post-ablation	10	4,240.3 ± 770.9^**^	447.1 ± 83.5^**^	21.3 ± 4.5^**^	253.7 ± 51.0^**^
2 d post-ablation	10	3,080.0 ± 365.1^**^	912.2 ± 394.5^**^	10.6 ± 1.1^*^	216.2 ± 43.8^**^
3 d post-ablation	10	2,921.9 ± 1,078.9^**^	2,824.1 ± 667.8^**^	13.5 ± 3.0^*^	408.8 ± 26.2^**^
1 w post-ablation	10	1,290.6 ± 697.5^**^	1,638.6 ± 974.9^**^	28.1 ± 3.5^*^	322.2 ± 188.4^**^
2 w post-ablation	10	484.6 ± 57.2^*^	236.8 ± 56.5	18.8 ± 5.0^**^	71.0 ± 4.7^*^
4 w post-ablation	6	763.6 ± 554.7^*^	236.2 ± 177.3	13.5 ± 4.7	62.5 ± 20.1^*^

CK, creatine kinase; CK-MB, creatine kinase–MB isoenzyme; AST, aspartate aminotransferase; LDH, lactate dehydrogenase.

* *P* < 0.05,

** *P* < 0.01 vs. sham group.

### Ablated left ventricular epicardium gross changes

**Figure 2:**
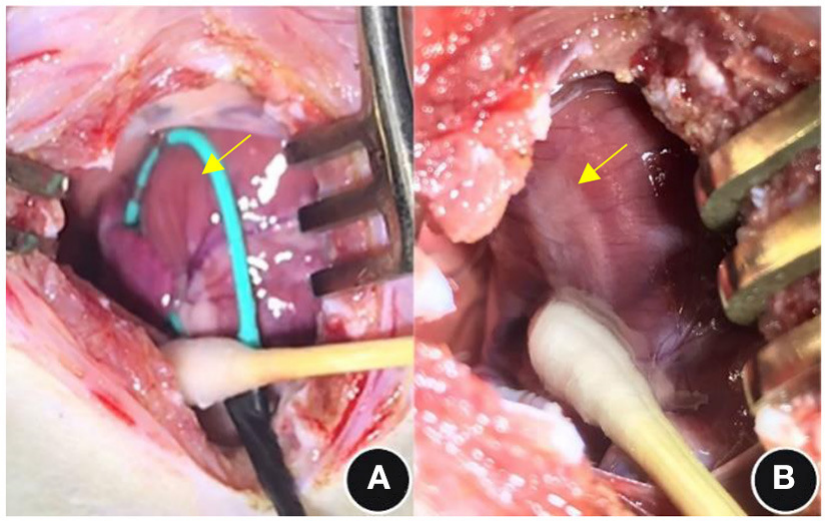
General observations before and immediately after PEF ablation. **(A)** Pre-ablation and **(B)** post-ablation; see the yellow arrows. PEF, Pulsed electric field.

The borders of the ablation area were clear after PEF ablation, and the edematous process started with obvious whitening see Figure 2.

### Myocardial histopathology after PEF ablation

**Figure 3:**
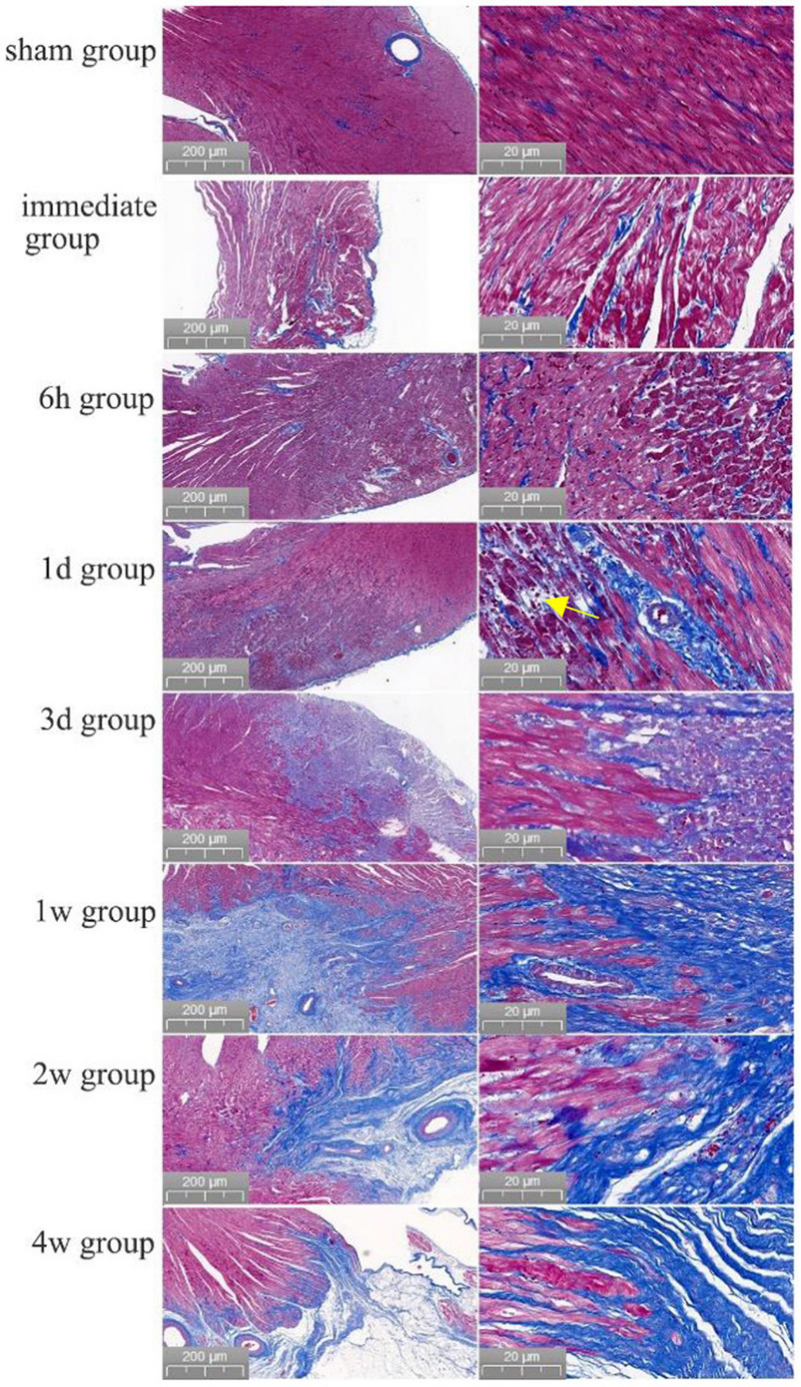
Evolution of myocardial necrosis at different time periods before and after pulsed ablation (Masson staining). In the preoperative heart, the myocardium tissue were neatly arranged and tightly packed. Immediately after the operation, it was obvious that the ablation boundary was clear, the myocardium tissue were swollen, and the cell gap became larger. Then, 6 h post-ablation, the boundary of PEF ablation was clearer, the myocardial cells were swollen, and the cell gap became larger; besides, the myocardial bundle was broken into myocardial fragments, and red blood cells appeared in the myocardial cell gap. At 1 day post-ablation, the ablation boundary was still clear, and the cardiomyocytes had begun to atrophy with a large frequency of erythrocyte infiltration (shown by yellow arrows). At 3 days post-ablation, the ablation boundary was clear, and the cardiomyocytes were atrophied and necrotic, with clustered nuclei and aggregated fibrous structures. At 1 week post-ablation, the ablation boundary was clear, most of the myocardial tissue in the ablation area had disappeared, the structure was intact with fibrotic replacement, and the vascular structure was intact without damage. At 2 weeks, the ablation boundary was clear, the myocardial cells in the ablation area had basically disappeared with fibrous connective tissue replacement, and the vascular structure was intact. After 4 weeks, the ablation boundary was clear, the myocardium tissue in the ablation area had almost completely disappeared, the fibrous structure was intact, and the vascular structure was intact.

**Figure 4:**
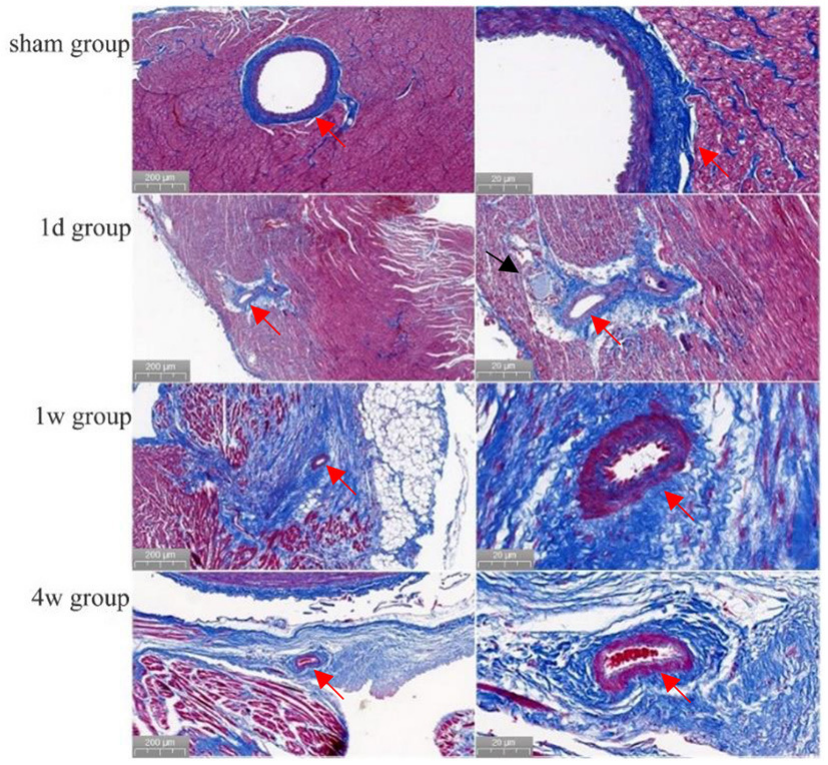
Morphology of cardiac vessels and nerves in the ablation area (Masson staining). Preoperatively, the endothelial cells of the arterial vessels were neatly and closely arranged with well-defined layers. Then, at 1 day, 1, and 4 weeks after ablation, no abnormal changes had occurred in the arterial vessels or endothelial cells (shown by red arrows). Also, at 1 day after ablation, the nerve structure was intact (shown by black arrows).

The boundary between the ablated area and normal myocardial tissue was always clear from immediately after PEF ablation to 4 weeks after PEF ablation. Swelling of myocardial cells was seen immediately after PEF ablation, and erythrocyte infiltration started after 6 h. After 1 day, the cells started to atrophy and exhibit necrosis; after 3 days, fibrotic replacement of the necrotic area was obvious. By 4 weeks, the myocardial cells had been completely replaced by fibrosis. The vascular structure in the ablation area remained intact (Figure 3). Myocardium tissue in the ablation area completely replaced by fibrous connective tissues 1–2 weeks after PEF ablation, and then completely replaced by fibrous connective tissues 4 weeks later. The vascular and nerve structures in the PEF ablation area remained intact (Figure 4).

### Analysis of myocardial cell apoptosis

**Figure 5:**
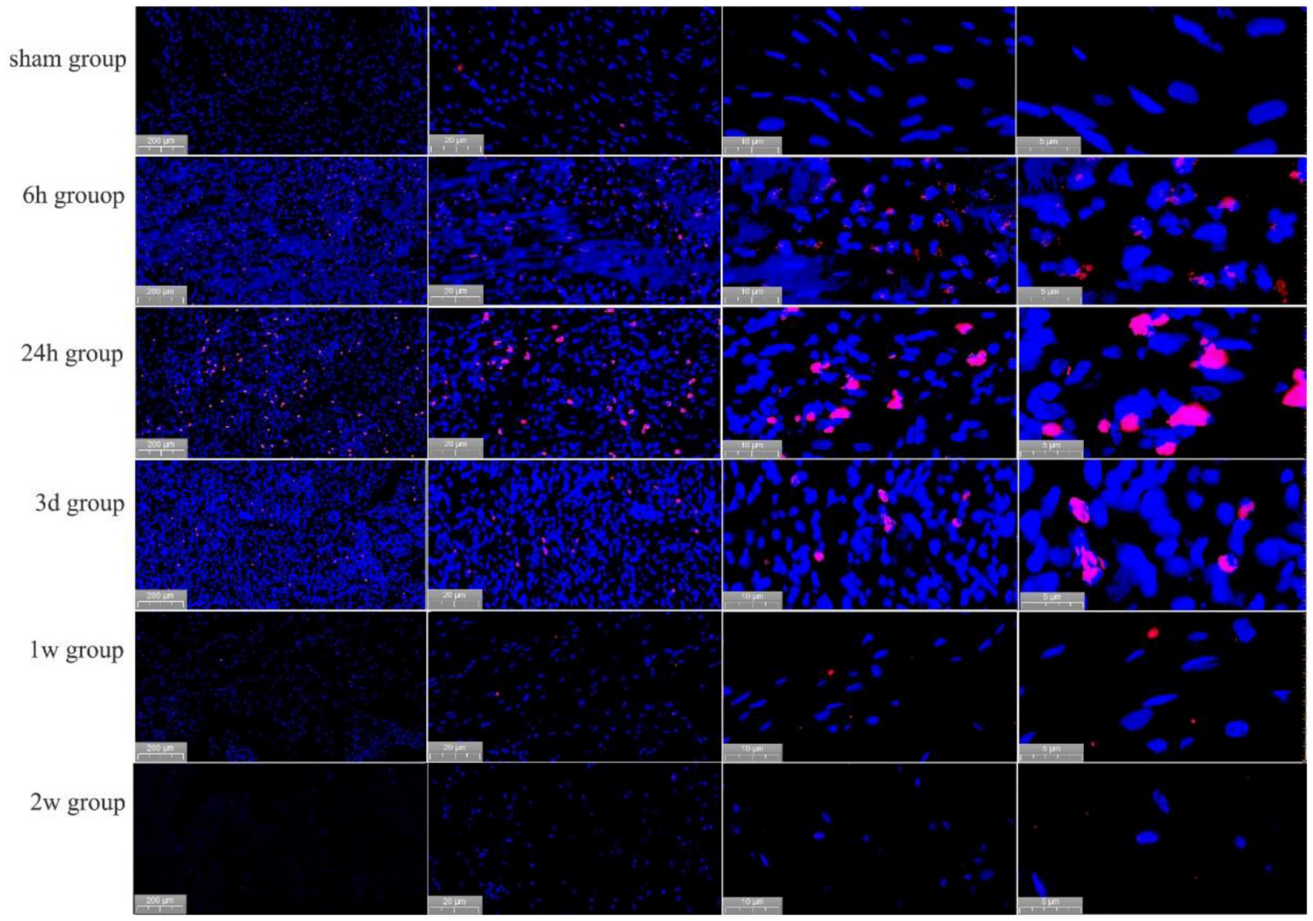
Apoptosis of cardiomyocytes, as detected by TUNEL staining, at different time points in the PEF ablation region. Immediately after ablation, no apoptotic cells were generated; at 6 h after ablation, apoptotic cardiomyocytes were seen; at 24 h after ablation, significant apoptotic cells were seen; at 3 days after ablation, a significant decrease in apoptotic cells was observed; and at 1 ~ 2 week after ablation, no apoptotic cells were seen. Positively apoptotic cells are cells with red-stained nuclei.

In the post-ablation immediate group, the apoptosis rate was 1.2 ± 1.3%, and no significant apoptosis occurred. In the 6 h postoperative group, cardiomyocytes apoptosis occurred significantly, and the apoptosis rate was 33.6 ± 5.9% (*P* < 0.01). In the 1d postoperative group, the apoptosis rate of cardiomyocytes reached a peak of 45.2 ± 7.6%, i.e., a 37.7-fold increase (*P* < 0.01). In the 3 d postoperative group, the apoptotic cells were significantly reduced, and the apoptosis rate was 27.0 ± 6.2% (*P* < 0.01). In the 1 w postoperative group, the apoptosis rate of cardiomyocytes was not significant, with an apoptosis rate of 1.8 ± 1.3% (*P* > 0.01), almost no apoptotic cells were seen 2 w later see Figure 5.

## Discussion

The basic biomedical engineering principles of PEF ablation are to release high electric-field energy in a short period of time at the microsecond and nanosecond levels by forming a local high-voltage difference through electrodes and to precisely deliver the electric field energy to the target area of the heart from the anatomically conformal design of the ablation catheter electrodes. PEF ablation increases cell membrane permeability through membrane electroporation, causing cell death (6). PEF ablation of cardiac myocytes is based on tissue resistance specificity, which determines the tissue PEF threshold differentiation, with the lowest ablation threshold of 400 V for cardiac myocytes, therefore prioritizing myocardial ablation, with complete cell death in the ablation area and less damage or even intact preservation of adjacent tissues such as blood vessels, nerves, and the esophagus in and around the ablation area (7). PEF ablation has brought about a change in the ablation catheter and ablation concept and is expected to be a new source of energy for arrhythmia ablation therapy, and the current findings suggest that this is a highly promising direction for clinical application (8). PEF ablation creates a larger and more homogeneous range of acute injury, which is beneficial in preventing decreased atrial/ventricular muscle compliance and in preserving atrial reserve and ejection function (9).

The PEF ablation system used in this research is based on a PEF ablation system that has been successfully developed for tumor ablation, and the PEF ablation instrument is characterized by its miniaturization and unique ablation method. It boasts the following innovations: (1) independent research and development of a chip semiconductor were performed, with independent intellectual property rights of an ultra–high-voltage, ultra–short-pulse generator; (2) being different from existing PEF electrical parameters, PEF waveform is sharper with a higher output energy; (3) the use of a cardiac synchronization control circuit in the ablation of the discharge in the non-phase effectively reduces the risk of induced malignant arrhythmia during the ablation; (4) the development and production of a polymer material with high self-adaptivity; and (5) a highly self-adaptive bendable ablation catheter made of polymer material was developed and produced, realizing the safety of insulation and a convenient operation. PEF ablation parameters and different treatment conditions correspond to induce biophysical dose effects that affect the cell death pathway and are key to optimizing clinical treatment protocols (10).

In the present study, it is clear that there is a dynamic evolution of the myocardial enzyme profile due to myocardial cell necrosis caused by PEF ablation. The evolution of CK and CK-MB values, which are highly specific for myocardial necrosis, was similar to that of acute myocardial infarction, while the evolution of AST and LDH values, which are less specific for myocardial necrosis, fluctuated and needs to be further explored. In this study, after PEF ablation in the rabbit left ventricular myocardium, the boundary between the ablated area of PEF and normal myocardial tissue was always clear, which is the basis of accurate ablation of arrhythmias; combined with Masson staining and TUNEL staining analysis of the ablation and peripheral areas, swelling of myocardial cells was observed immediately after PEF ablation, and myocardial cells entered into apoptosis 6 h after ablation, beyond which point the cell gap became larger and myocardial bundles broke into myocardial segments. The myocardial cell gap was accompanied by the appearance of red blood cells, and the process of atrophy and necrosis began, peaking after 1 day. Eventually, the apoptotic process in the ablation region ended after 3 days. The apoptotic necrotic region in the ablation region was replaced by fibrosis from 1 to 3 days. Most of the myocardial cells disappeared after 1 week, basically disappeared after 2 weeks, and almost completely disappeared after 4 weeks. The myocardial cells were replaced by fibrosis, the ablation boundary was clear, and no island-shaped surviving myocardium was observed. The regional vascular and neural structures were intact, and the myocardial cell death process was a necrotizing apoptotic process. The molecular biological mechanism of necrotizing apoptosis of myocardium tissue due to PEF ablation needs to be further investigated (11, 12).

## Conclusion

We performed PEF ablation on rabbit left ventricular epicardium using our self-developed cardiac PEF ablation system with the biphasic voltage set to ±800 V, cardiomyocytes showed apoptotic process and dyed, at least partially, through a secondary necrosis, and the ablation boundary was clear. The ablation area was later replaced by structurally intact fibroblasts with no island myocardium tissue visible, and the blood vessels and nerves in the ablation area were not affected. The clinical application of our PEF ablation system is based on this study.

## Data availability statement

The raw data supporting the conclusions of this article will be made available by the authors, without undue reservation.

## Author contributions

XC and XZ conceived and designed the study. YC, BW, and GQ collected data. ZZ and XC analyzed the data. ZZ and LH drafted the article. The manuscript was approved by all the above authors.

## Funding

This work was supported by the National S&T Major Project of China (2018ZX10301201), NSFC (82027803, 82070516, and 82102183), and Hangzhou Health Commission Medical Enterprise Joint Project (2021JWCY239).

## Conflict of interest

The authors declare that the research was conducted in the absence of any commercial or financial relationships that could be construed as a potential conflict of interest.

## Publisher's note

All claims expressed in this article are solely those of the authors and do not necessarily represent those of their affiliated organizations, or those of the publisher, the editors and the reviewers. Any product that may be evaluated in this article, or claim that may be made by its manufacturer, is not guaranteed or endorsed by the publisher.
